# Non-junctional role of Cadherin3 in cell migration and contact inhibition of locomotion via domain-dependent, opposing regulation of Rac1

**DOI:** 10.1038/s41598-020-73862-y

**Published:** 2020-10-15

**Authors:** Takehiko Ichikawa, Carsten Stuckenholz, Lance A. Davidson

**Affiliations:** 1 Department of Bioengineering, University of Pittsburgh, 3501 Fifth Avenue, 5059-BST3, Pittsburgh, PA 15260 USA; 2 Nano Life Science Institute (WPI-NanoLSI), Kanazawa University, Kanazawa, 920-1192 Japan; 3 Department of Developmental Biology, University of Pittsburgh, Pittsburgh, PA 15260 USA; 4 Department of Computational and Systems Biology, University of Pittsburgh, Pittsburgh, PA 15260 USA

**Keywords:** Cellular motility, Collective cell migration, Mesenchymal migration, Cadherins, Small GTPases

## Abstract

Classical cadherins are well-known adhesion molecules responsible for physically connecting neighboring cells and signaling this cell–cell contact. Recent studies have suggested novel signaling roles for “non-junctional” cadherins (NJCads); however, the function of cadherin signaling independent of cell–cell contacts remains unknown. In this study, mesendodermal cells and tissues from gastrula stage *Xenopus laevis* embryos demonstrate that deletion of extracellular domains of Cadherin3 (Cdh3; formerly C-cadherin in *Xenopus*) disrupts contact inhibition of locomotion. In both bulk Rac1 activity assays and spatio-temporal FRET image analysis, the extracellular and cytoplasmic Cdh3 domains disrupt NJCad signaling and regulate Rac1 activity in opposing directions. Stabilization of the cytoskeleton counteracted this regulation in single cell migration assays. Our study provides novel insights into adhesion-independent signaling by Cadherin3 and its role in regulating single and collective cell migration.

## Introduction

Cadherins are transmembrane, calcium-dependent adhesive molecules that mechanically connect the actin cytoskeleton of one cell to the actin cytoskeleton of its neighboring cells^1, 2^. Additionally, cadherins signal into the cytosol when their extracellular domains interact with other transmembrane proteins, for example, epidermal growth factor receptor (EGFR) or P2Y_2_ receptor (P2Y_2_R). Cytosolic cadherin domains interact with other cytosolic proteins, such as p120-catenin (p120), β-catenin, and α-catenin^3–6^. However, these signaling pathways have been studied under conditions where cells maintain cell–cell contact^7, 8^. By contrast, recent studies have focused on the role of unbound cadherins, so-called “non-junctional” cadherins (NJCads), which also function as signal transducers^1, 2^. Zaidel-Bar and co-workers showed that non-junctional Cdh1 (E-cadherin) clusters inhibit the activity of RhoA and myosin ll and impede cortical actin flows in *C. elegans* zygotes^1^. Walsh and co-workers demonstrated that an extracellular-truncated form of Cdh2 (N-cadherin) alters the collective migratory behaviors of facial branchiomotor neurons in zebrafish embryos^2^. In both cases, these novel roles of cadherin are cell-autonomous and are not the result of the loss of cell–cell junctions. However, intracellular pathways that mediate NJCads signaling are poorly understood.

Truncation mutants of cadherins, i.e., deletions of the extracellular-domain (ΔE-cdh) or the cytosolic-domain (ΔC-cdh) have been frequently used in studies of coordinated cell behaviors and collective cell migration during embryogenesis^9, 10^. Expression of either ΔE-cdh2 or ΔC-cdh2 in *Xenopus* embryos caused lesions in the ectoderm, e.g. gaps in the surface epithelium, but embryos developed normally until mid-gastrulation^11^. Similar results were reported using ΔE-cdh1^12^. Expression of ΔC-cdh3 (Cdh3; here the alloallele Cdh3.S, previously known as C-cadherin) in *Xenopus* embryos induced a delay in blastopore closure while leaving anterior patterning intact^13^. In all cases, the mesendoderm expressing truncation-mutant cadherin retained the ability to migrate, but failed to close the blastopore correctly.

Contact inhibition of locomotion (CIL) is the behavior of a cell that causes the cell to stop or change its direction after colliding with another cell, i.e. with a confronting cell^14–16^. After contact, the leading-edge of the protrusion collapses near the contact site, and a new leading-edge forms at a different position on the cell, causing the cell to move in a different direction^17, 18^. When CIL fails, the cell continues to migrate through the confronting cell with little or no change in direction. To date, several signal components, including RhoA, Rac1, ephrin receptor (Eph), platelet-derived growth factor (PDGF), and non-canonical Wnt pathways, have been implicated in regulating CIL^19–23^. As for cadherins, Cdh1 and Cdh11 repress and Cdh2 activates CIL in neural crest cells of *Xenopus* embryos^24–26^. However, it is still unclear whether these effects are the result of cadherin-cadherin binding during cell-adhesion or reflective of their non-junctional role.

Using motility assays, we demonstrate that defects in CIL occur cell-autonomously when cells express ΔE-cdh3. Moreover, expression of ΔE-cdh3 and ΔC-cdh3 produces opposite effects on single-cell directionality and Rac1 activity. Rac1 activity in cells expressing ΔE-cdh3 decreases, but increases in cells expressing ΔC-cdh3. Live-cell imaging of Rac1 activity using a *Xenopus*-optimized Raichu FRET reporter highlights the spatial and temporal dynamics of Rac1 throughout CIL and how the Cdh3 mutants disrupt directionality and cytoskeletal stability in CIL. Our findings support a model where NJCads, acting through Rac1 and cytoskeletal stability, regulate dynamic changes in cell orientation and persistence that mediate CIL and regulate collective cell movements.

## Results

### Methods for evaluating the role of non-junctional cadherins (NJCads) in CIL during collective tissue movements and during single-cell migration

**Figure 1:**
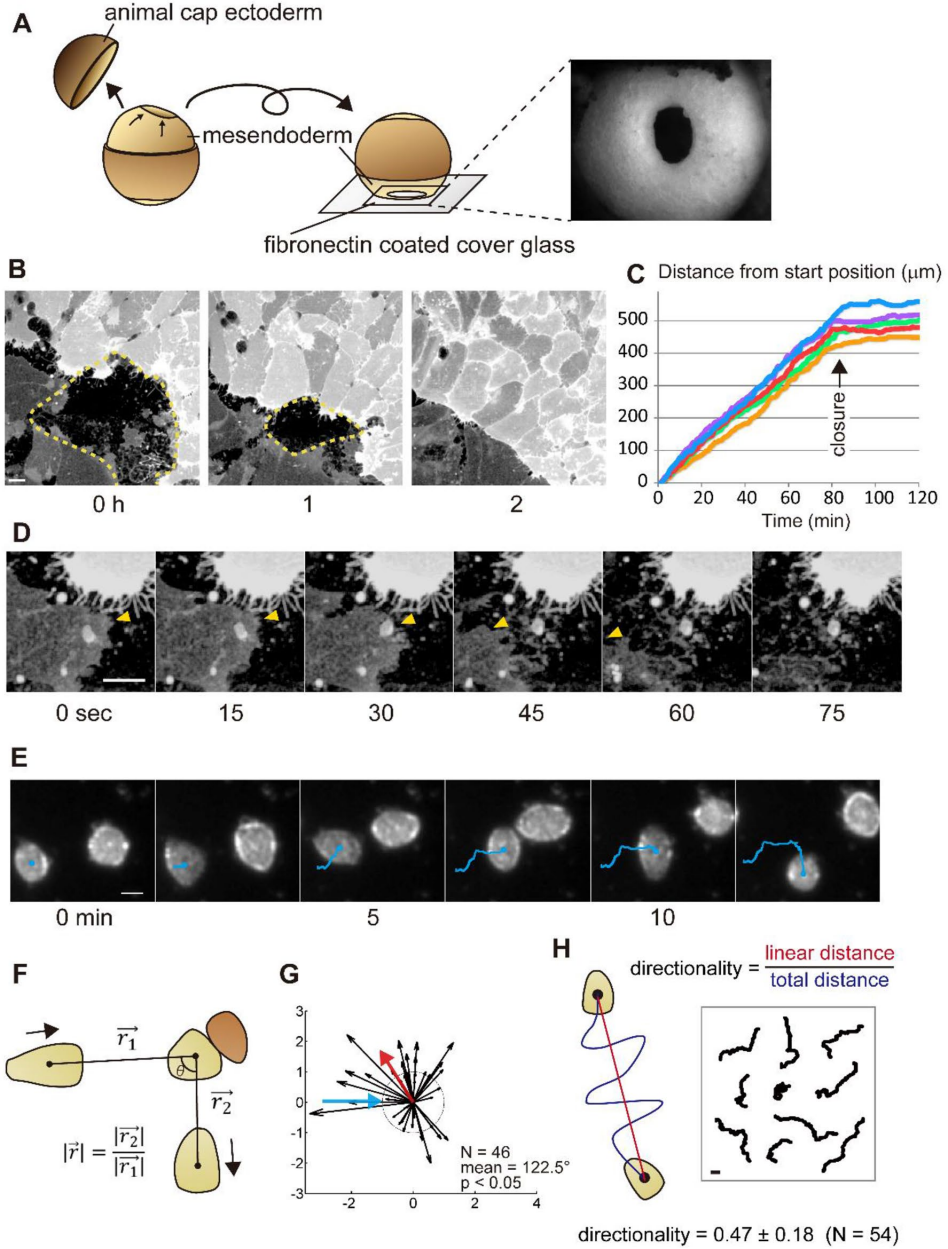
Cell migration assays using *Xenopus* gastrula stage embryonic mesendoderm; contact inhibition of locomotion (CIL) in collective migration, CIL in single migratory cells, and directionality of single motile cells. (**A**) Schematic of intravital imaging of mesendoderm closure in *Xenopus* embryo from stage 11.5. Animal cap ectoderm was removed, and the lip and outer surface of the mesendodermal mantle was placed in contact with a fibronectin-coated cover glass. The right-side image shows the mesendoderm mantle observed with a stereomicroscope. (**B**) Frames from a confocal time-lapse showing closing mesendoderm mantle expressing membrane-targeted GFP (dotted lines indicate the boundaries). A difference in the expression level of GFP indicates the different origins of opposing sides. (**C**) Progressive rates of closure from five embryos. Progress of the leading edge from the start time point to closure at each time point is shown. The arrow indicates the time of the collision. After the collision, cell migration stops. (**D**) Frames from a representative sequence showing lamellipodia retraction. The leading-edge on the darker cell is indicated by yellow arrowheads; the leading edge lamellipodia retracts after touching the brighter opposing cell. (**E**) Representative frames from a brightfield time-lapse sequence of colliding single mesendodermal cells. The trajectories are shown with blue lines. WT mesendodermal cells change migration direction after the collision. (**F**) The geometry of single-cell CIL kinetic analysis with a migratory cell (light brown) and an opposing cell (dark brown). (**G**) Vector plot of a set of wild type (WT) cell collisions (blue arrow—incoming cell; red arrow—mean angle post-collision; dotted circle radius 1—same velocity before and after collision). The mean value of the angle taken by the departing cell (incident angle = 0°) is calculated for a number of collisions (N). Statistical significance is calculated for a circular distribution of post-collision angles (p). (**H**) Schematic for measurement of directionality (left) and tracked paths of single migrating cells (right). All scale bars are 20 µm. The illustrations were drawn using Adobe Illustrator Version 24.1.1 (https://www.adobe.com/products/illustrator.html).

To investigate the function of NJCads in CIL, we adapted three assays: (1) CIL in collective migration, (2) CIL in single-cell migration, and (3) the directionality of single migrating cells. To assess CIL in collective migration, we observed the closure of the mantle-shaped mesendoderm of *Xenopus laevis* embryos at the end of gastrulation^27^. Mesenchymal mesendodermal cells migrate on the underside of the blastocoel roof toward the animal pole of the embryo to enclose the blastocoel^28–30^. Mesendoderm movements can be recorded using intravital microscopy in minimally manipulated embryos where a portion of the animal cap ectoderm is removed; the resulting embryo is positioned so that the mesendoderm is placed on a fibronectin-coated cover glass (Fig. 1A)^27^. Mesendoderm cells in these preparations extend large lamellipodia at the leading edge, and the cells elongate as the ring of leading-edge cells converges. CIL can be observed when lamellipodia of leading mesendodermal cells retract after contacting the opposing mesendodermal cells (arrowheads in Fig. 1D, Movie 1). Transient retractions of lamellipodia do not immediately arrest collective migration; however, collective migration ceases after cells at the leading edge collide with cells on the opposing margin (Fig. 1B,C). Migrating sheets of mesendoderm exhibit CIL similar to CIL observed during collective migration of neural crest cells^20, 23, 25^.

Since junctional cadherins might obscure the role of NJCads during collective migration, we adapted an assay to quantify CIL in single mesendodermal cells. We dissociated mesendoderm into single cells, plated them on fibronectin-coated glass, and tracked single-cell collisions in time-lapse sequences (Fig. 1E)^31–33^. We quantified cell–cell interactions by comparing the change in direction and velocity before and after collision (Fig. 1F)^20, 23, 34^. As expected, we found that wild type (WT) mesendodermal cells change direction after collision (Fig. 1G, Movie 2, and Table S1; N = 46, mean = 122.5°, *p* < 0.05), indicating that single mesendoderm cells retain the capacity for CIL.

To eliminate the potential role of cadherin-cadherin junctional interactions, we next tested the behaviors of single migrating cells. We prepared single mesendodermal cells as described above and collected one-hour time-lapse sequences as they migrated without cell–cell contact (Fig. 1H). We analyzed the tracks of single cells over this time to determine their average directionality ratio, d/D, where D is the total path length between start and end, d is the linear distance from start to end, and found the directionality of single WT mesendoderm cells is 0.47 ± 0.18 (Fig. 1H, mean ± SE, N = 54). Thus, equipped with assays for tissue- and cell-scale CIL and single-cell migration, we turned to investigate the role of NJCads on collective migration.

### Truncation mutant cadherins ΔE-cdh3 and ΔC-cdh3 alter CIL and directional migration in mesendoderm cells

**Figure 2:**
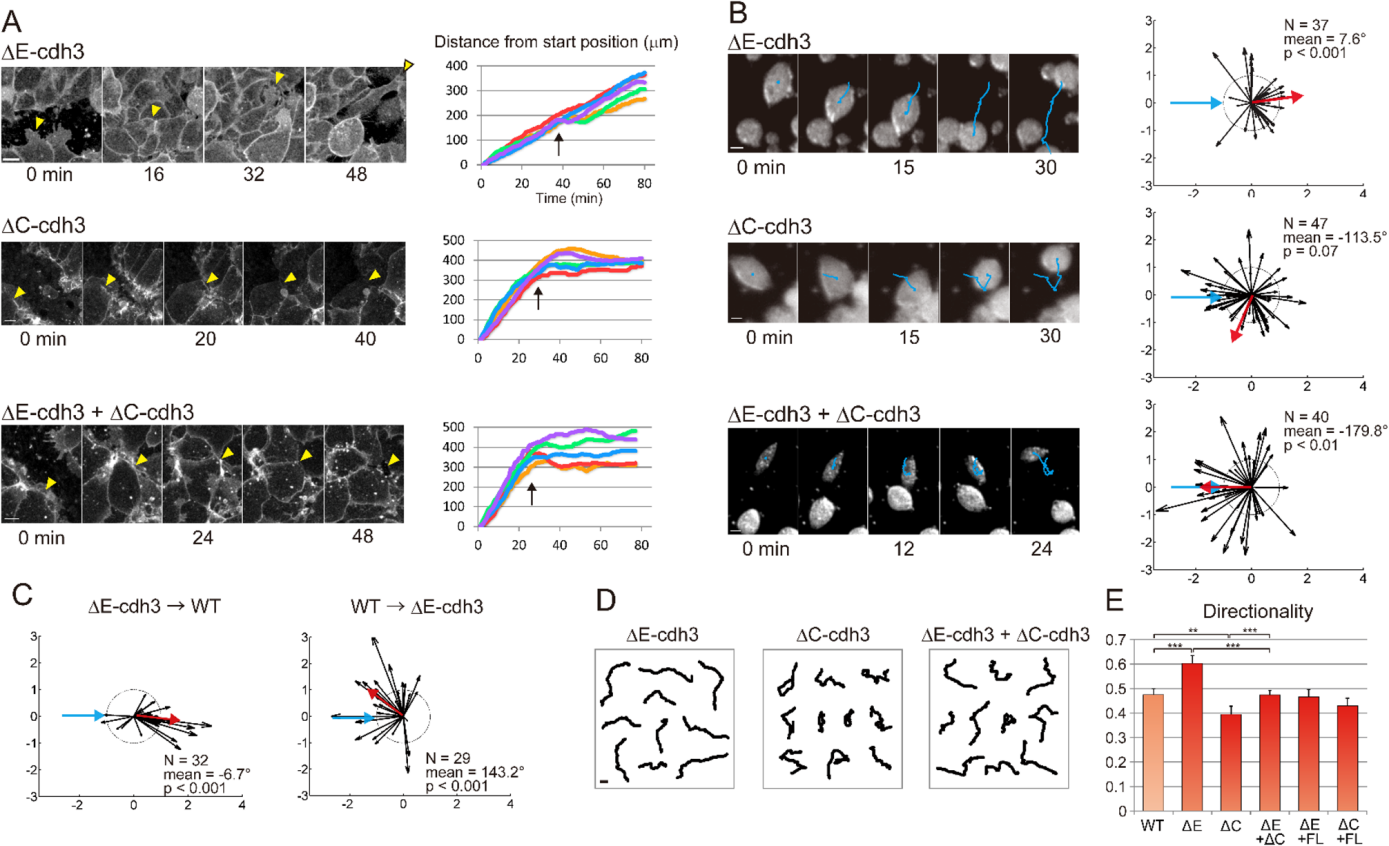
Truncation mutants of Cdh3 independently regulate collective CIL, single cell CIL, and single cell persistence. (**A**) Frames from a representative confocal time-lapse sequence (left; leading edge, yellow arrowheads) of membrane-targeted GFP expressing mesendoderm mantle closure in embryos expressing extracellular truncated Cdh3 (ΔE-cdh3), cytoplasmic domain truncated Cdh3 (ΔC-cdh3), and ΔE-cdh3 + ΔC-cdh3. Positions of leading edge mesendoderm movements during closure (including sequences shown at left, n = 5; arrows indicate time of collision). (**B**) Collisions of ΔE-cdh3, ΔC-cdh3, and ΔE-cdh3 + ΔC-cdh3 expressing single cells (left; trajectories shown in blue). Angle followed by cells after collision (right; mean angle, red; ΔE-cdh3, N = 37, mean = 7.6°, *p* < 0.001; ΔC-cdh3, N = 40, mean = − 179.8°, *p* < 0.01; ΔE-cdh3 + ΔC-cdh3, N = 47, mean = − 113.5°, *p* = 0.07). (**C**) Collisions between ΔE-cdh3 expressing cells and WT cells. Left: Summary of collisions of ΔE-cdh3 expressing cells into WT cells (N = 32, mean = − 6.7, *p* < 0.001). Right: Summary of single cell collisions of WT cells into ΔE-cdh3 expressing cells (N = 29, mean = 143.2, *p* < 0.001). (**D**) Single cell trajectories over 1 h without collision of ΔE-cdh3, ΔC-cdh3 and ΔE-cdh3 + ΔC-cdh3 expressing cells. (**E**) Directionality of single cell migration of ΔE-cdh3 (ΔE), ΔC-cdh3 (ΔC), ΔE-cdh3 + ΔC-cdh3 (ΔE + ΔC), ΔE-cdh3 + full length of Cdh3 (FL-cdh3) (ΔE + FL), ΔC-cdh3 + FL-cdh3 (ΔC + FL). All scale bars are 20 µm.

Since Cdh3 is the only cadherin expressed in mesendoderm during gastrulation^35^, we used truncation mutants of Cdh3, ΔE-cdh3, and ΔC-cdh3, lacking extracellular and cytoplasmic domains, respectively^36^, to modulate the function of Cdh3. We confirmed the expression of exogenous ΔE-cdh3 or ΔC-cdh3 by Western blot (Fig. S1). Surprisingly, when we tested tissue CIL we found mesendoderm sheets expressing ΔE-cdh3 did not stop after collision (Fig. 2A top panel, Movie 3). Instead, leading-edge mesendoderm cells expressing ΔE-cdh3 ran over, or invaded, opposing mesendoderm in a fashion resembling the behavior of neural crest cells defective in CIL^20, 23^, or cell types lacking CIL behaviors^34^. By contrast, mesendoderm expressing ΔC-cdh3 alone or ΔE-cdh3 with ΔC-cdh3 exhibited normal CIL and stopped migrating after the collision, akin to WT mesendoderm (Fig. 2A).

To test the effect of truncated cadherins on CIL between single-cell pairs, we carried out collisions between single mesendodermal cells, each expressing ΔE-cdh3 and found that they too lacked CIL (Fig. 2B, Movie 4; N = 37, mean = 7.6°, *p* < 0.001), whereas two single cells expressing ΔC-cdh3 retained CIL when colliding with each other. Co-expression of ΔE-cdh3 and ΔC-cdh3 partially rescued the deficiency of CIL caused by expression of ΔE-cdh3 alone (Fig. 2B, Movie 5, 6; N = 47, mean = -113.5°, *p* = 0.07). Defects in CIL caused by expressing ΔE-cdh3 were also partially rescued by co-expressing full-length Cdh3 (FL-cdh3, Fig. S3A). Importantly, we confirmed that CIL was cell-autonomous, with CIL occurring after WT cells collided with ΔE-cdh3 cells and not occurring when ΔE-cdh3 expressing cells collided with WT cells (Fig. 2C). Furthermore, we measured CIL between WT cells and Cdh3 conjugated beads, because cadherin-cadherin junctional binding to the opposite cell alone might change the activity of small RhoGTPases^7, 37–39^ and that subsequent defects of CIL in ΔE-cdh3 cells might be due to the loss of junctional cadherin-cadherin binding (Fig. S2). The results of CIL analysis with Cdh3 conjugated beads indicate CIL does not require the formation of junctions between extracellular domains of Cdh3. Thus, our analyses at the tissue- and single-cell levels show that CIL can be regulated cell-autonomously by Cdh3 truncation mutants, supporting a role for NJCads in CIL.

Since directional cell migration contributes to the operation of CIL, we sought to test the effects of Cdh3 mutants on the migration directionality in the absence of cell–cell contact^40–42^. We were surprised to find ΔE-cdh3 expressing cells were 27% more directional than WT cells (Fig. 2D,E, and Table S2; in contrast to WT trajectories shown in Fig. 1H), and that conversely, ΔC-cdh3 expressing cells exhibited 17% lower directionality than WT. Directionality was also restored to wild type levels when full-length Cdh3 was co-expressed with either ΔE-cdh3 and ΔC-cdh3. These results indicate that the extracellular and cytoplasmic domains of Cdh3 have the opposite effect on the persistence of cell motility and suggest intracellular and extracellular Cdh3 domains might activate and inhibit, respectively, the same target pathway.

### Expression of ΔE-cdh3 decreases while ΔC-cdh3 increases Rac1 activity

**Figure 3:**
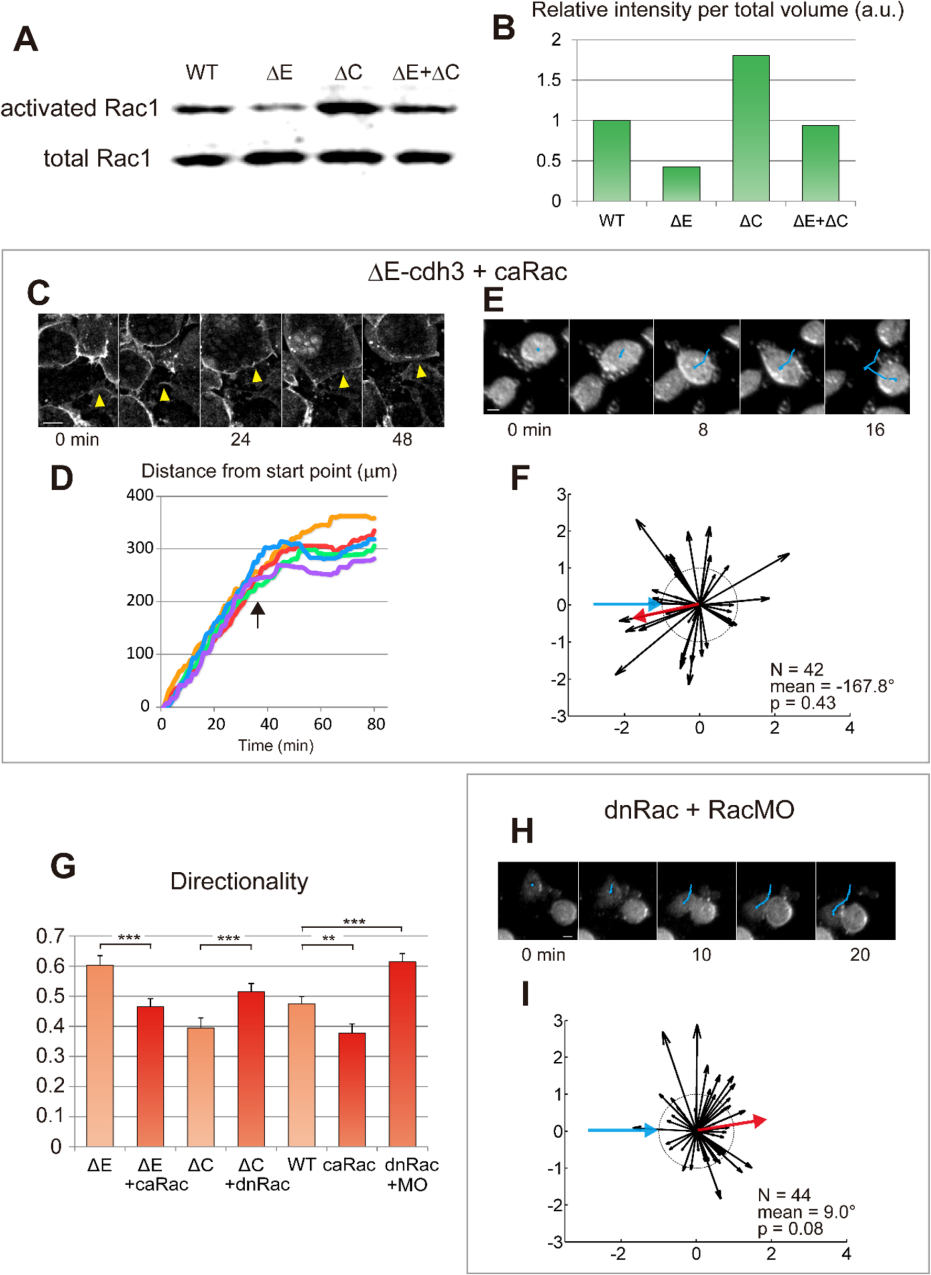
Truncated cadherins modulate Rac1 activity responsible for single cell CIL, collective CIL, and single cell persistence. (**A**) Rac1 activity is modulated from WT levels by ΔE-cdh3 (ΔE), ΔC-cdh3 (ΔC), and ΔE-cdh3 + ΔC-cdh3 (ΔE + ΔC). (**B**) Rac1 activity of (**A**) normalized to WT levels. Rac1 activity in ΔE-cdh3 and ΔC-cdh3 expressing cells was 0.4- and 1.8-fold of WT levels. ΔE + ΔC activity was not significantly different from WT levels. Full-length blots are presented in Figure S7A. (**C**) Mesendoderm closure in embryos co-injected with ΔE-cdh3 + constitutively active form of Rac1 (caRac). (**D**) Time-courses of mesendoderm closure from five embryos. (**E**) Single cell collisions of ΔE-cdh3 + caRac expressing cells. (**F**) Summary of collisions of ΔE-cdh3 + caRac expressing cells (N = 42, mean = − 167.8°, *p* = 0.43). (**G**) Directionality of single cell migrations of ΔE-cdh3 + caRac (ΔE + caRac), ΔC-cdh3 + dominant negative form of Rac1 (dnRac) (ΔC + dnRac), caRac and dnRac + Rac1 morpholino oligomers (RacMO) (dnRac + RacMO) compared with ΔE-cdh3 (ΔE), ΔC-cdh3 (ΔC) and WT, respectively (light red for comparison from Fig. 2E). (**H**) Single cell collisions of dnRac + RacMO co-injected cells. (**I**) Summary of collisions of dnRac + RacMO co-injected cells (N = 44, mean = 9.0°, *p* = 0.08). All scale bars are 20 µm.

The effect of mutant cadherins on single-cell persistence of migration is reminiscent of the effect of Rac1^40, 41, 43^ and suggests that Rac1 is inhibited in ΔE-cdh3 expressing cells and activated in ΔC-cdh3 expressing cells. To test this, we measured the activity of Rac1 in mesendoderm tissues isolated from ΔE-cdh3, ΔC-cdh3, and ΔE-cdh3 and ΔC-cdh3 co-expressing embryos^44^. Rac1 activity in ΔE-cdh3 expressing tissues was approximately 40% of WT levels, and Rac1 activity of ΔC-cdh3 expressing tissues was approximately 180% higher than WT (Fig. 3A,B, and Fig. S6). In tissues co-expressing ΔE-cdh3 and ΔC-cdh3, the activity was almost equal to WT tissues. Thus, ΔC-cdh3 or ΔE-cdh3 of Cdh3 activate and inhibit Rac1 activity in mesendoderm, respectively.

To test whether Rac1 serves as an effector of non-junctional Cdh3 in CIL, we assessed whether Rac1 activation or inhibition could rescue the CIL and cell motility changes induced by ΔE-cdh3 or ΔC-cdh3. We expressed ΔE-cdh3 together with a constitutively active form of Rac1 (caRac) and found that mesendodermal movement after closure and the defects in single-cell CIL caused by ΔE-cdh3 were partially rescued by co-expression with caRac1 (Fig. 3C–F; N = 42, mean =  − 167.8°, *p* = 0.43)^45^. Co-injection of ΔE-cdh3 with caRac and co-injection of ΔC-cdh3 with a dominant-negative form of Rac1 (dnRac) rescued both the high and low directionality of single migratory cells caused by ΔE-cdh3 and ΔC-cdh3, respectively (Fig. 3G and Table S2)^46^. Similarly, mild inhibition of Rac1 using a Rac1 inhibitor (20 nM NSC23766) also rescued the low directionality of ΔC-cdh3 expressing cells (Fig. S3B and C)^47^. These results indicate that changes in CIL and motility induced by ΔE-cdh3 and ΔC-cdh3 can be rescued by compensatory modulation of Rac1 activity.

We also tested whether modulated Rac1 activity alone could alter CIL and directionality. In order to reduce Rac1 activity as much as possible, we co-injected RNA encoding dnRac together with an antisense morpholino against endogenous Rac1 (RacMO) which is designed not to interfere with the translation of the dnRac construct (Fig. S4). Co-injection of dnRac and RacMO reduced Rac1 activity to 7% of WT (Fig. S4A, B). The inhibition of Rac1 activity by the co-injection of dnRac with RacMO showed partial inhibition of CIL and increased directionality (Fig. 3G–I; N = 44, mean = 9, *p* = 0.08). High concentrations of Rac1 inhibitor (300 µM NSC23766) showed similar results when applied to WT cells (Fig. S3C; N = 49, mean = − 11.5°, *p* = 0.17). caRac injection also resulted in low directionality (Fig. 3G). These results support a mechanism where Cdh3 controls CIL and cell migration by regulating Rac1 activity.

### Rac1 activated cell boundary domains are reduced in ΔE-cdh3 expressing cells and increased in ΔC-cdh3 expressing cells

**Figure 4:**
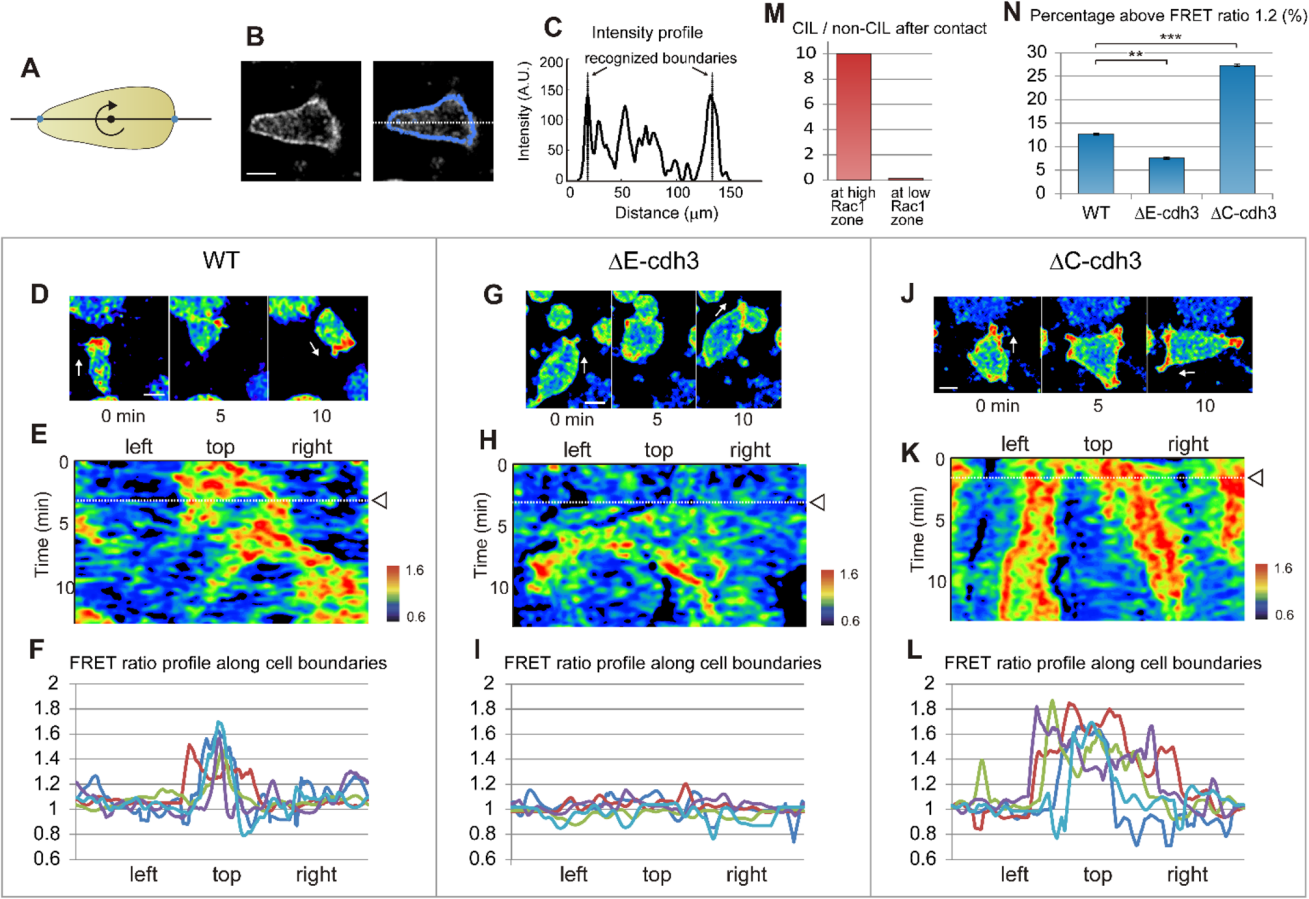
Truncated cadherins alter the spatial patterns of Rac1 activity during cell–cell collisions. (**A**) Schematic of the method for segmenting the cell membrane of migrating cells. The line passing through the center of the cell detects peaks on both sides in the YFP channel and rotates 360° by 1°. (**B**) Representative Rac1 biosensor FRET image (CFP channel, left) and segmented points for activity quantification (blue line, right). (**C**) The intensity profile of Rac1 activity along the white dotted line (**B**; cell boundary indicated). (**D**) Representative FRET images before, during, and after a typical cell–cell collision of single WT cell. Rac1 activity is shown in pseudocolor; white arrows indicate the directions of cell migrations. (**E**) Kymograph of Rac1 activity along the cell perimeter during a cell–cell collision with cell initially moving to the top of the frame in (**D**). The center of the x-axis indicates the front of the migrating cell prior to the collision. The arrowhead and dotted line indicate the time of the collision. After the collision, (**F**) FRET profile along cell perimeters of five typical cells. The center of the x-axis indicates the front of the migrating cell. (**G**) Time-course images before, during, and after a typical cell–cell collision of single ΔE-cdh3 expressing cell. (**H**) Kymograph during the cell–cell collision of ΔE-cdh3 expressing cell. (**I**) FRET ratio profile along cell perimeters of five typical cells. (**J**) Time-course images before, during, and after a typical cell–cell collision of single ΔC-cdh3 expressing cell. (**K**) Kymograph during the cell–cell collision of ΔC-cdh3 expressing cell. (**L**) FRET ratio profile along cell perimeters of five typical cells. (**M**) The frequency of WT CIL is high when the first contact occurs in a high Rac1 activity zone and low when contact occurs in a low Rac1 activity zone. (N = 77 contacting at high Rac1 zone, 9 contacting at low Rac1 zone). (**N**) Percentage of cell perimeter above ratio 1.2 along cell boundary in WT, ΔE-cdh3, and ΔC-cdh3 cells. WT: 12.6 ± 0.18 (mean ± SE)% (N = 57), ΔE-cdh3: 7.6 ± 0.22% (N = 50, *p* < 0.01), ΔC-cdh3: 27.3 ± 0.26% (N = 59, *p* < 0.001).

To investigate the spatial and temporal dynamics of Rac1 activity during cell–cell collisions and how these dynamics are altered in ΔE-cdh3 and ΔC-cdh3 expressing cells, we adapted the Raichu-Rac FRET biosensor^48, 49^ for use in *Xenopus*, and confirmed that the dynamic FRET signals reflect Rac1 activity in *Xenopus* mesendodermal cells (Fig. S5). To analyze the polarity of Rac1 activity, we quantified the FRET signal at cell membranes using custom image analysis code (Fig. 4A–C) in WT, ΔE-cdh3, and ΔC-cdh3 expressing cells during cell–cell collisions. In WT cells, Rac1 activity was high at the front of the migrating cell as reported previously (Fig. 4D–F)^50, 51^. As the cell collides with a stationary cell, Rac1 activity disappears from the contact site within two minutes, and increases at a different location. Following contact, the cell migrated away in the direction of the new site of Rac1 activity (Fig. 4D,E, and Movie 7). Interestingly, contacts initiated at sites of high Rac1 activity induced stronger CIL than contacts initiated in regions of low Rac1 activity (Fig. 4M). In ΔE-cdh3 expressing cells, Rac1 activity was lower throughout the cell and did not change during collisions (Fig. 4G–I, and Movie 8); as expected, cells continued to migrate in the same direction as prior to the collision. By contrast, Rac1 was activated at multiple locations along the cell periphery in ΔC-cdh3 expressing cells; CIL was unperturbed in these cells as migration ceased in the initial direction after collision (Fig. 4J–L, and Movie 9). To quantify the spatial pattern of high Rac1 activity along the cell periphery, we calculated the percentage of the cell’s boundary where the FRET signal ratio was higher than 1.2 (Fig. 4N). We found high Rac1 activity over 12.6 ± 0.2% of the periphery of WT cells (N = 57), 7.6 ± 0.2% of ΔE-cdh3 (N = 50, *p* < 0.01), and 27.3 ± 0.3% of ΔC-cdh3 (N = 59, *p* < 0.001). Live analysis of Rac1 activity revealed that CIL coincided with the transfer of Rac1 activity away from sites of contact and that spatial domains of high Rac1 activity were further restricted in ΔE-cdh3 and expanded in ΔC-cdh3 expressing cells.

### NJCads regulates cell migration through regulating cytoskeleton stability

**Figure 5:**
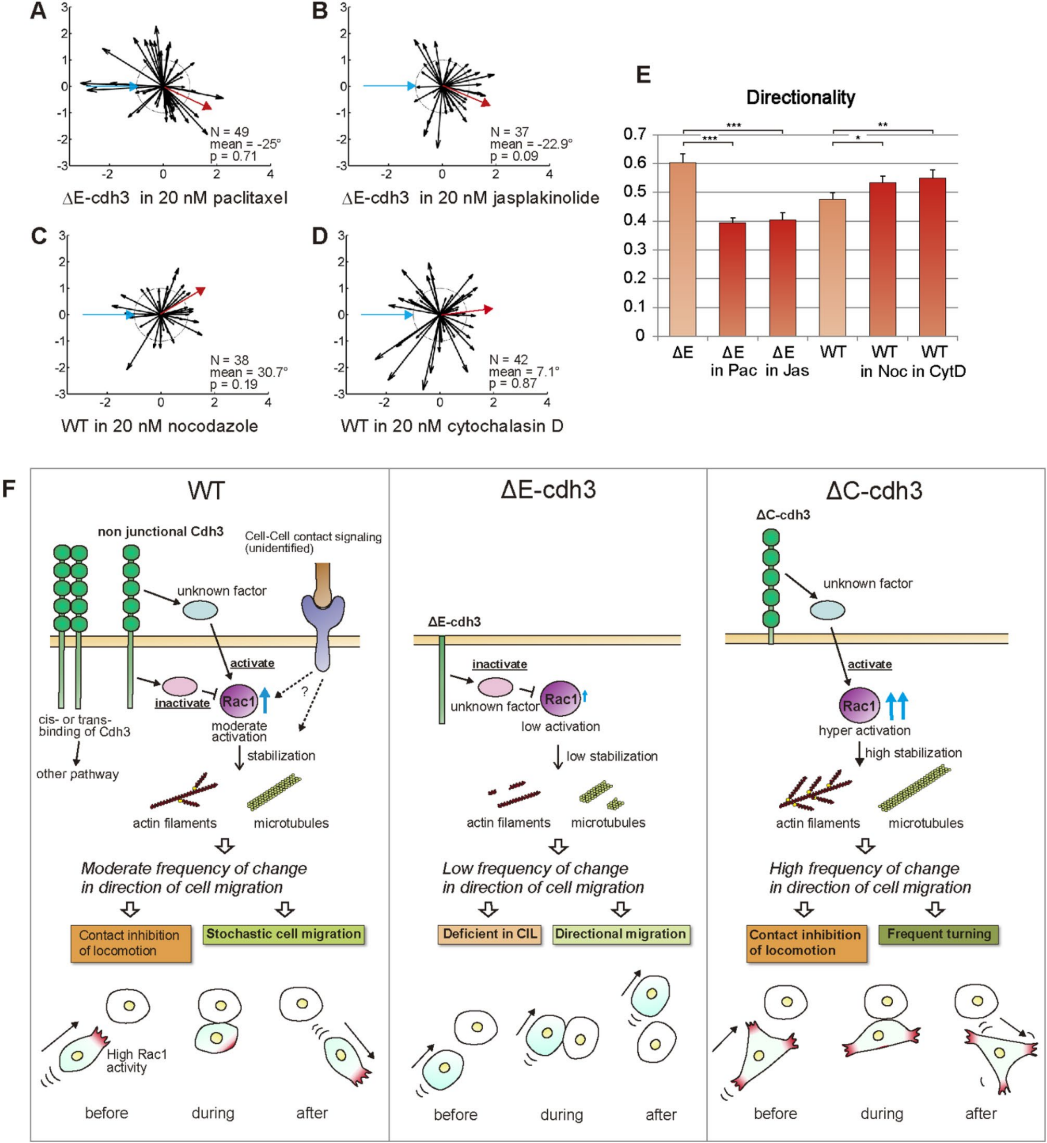
Effects of non-junctional cadherin3 on single-cell CIL and persistence of single-cell migration mitigated by changes in cytoskeletal stability. (**A**) Summary of collisions of ΔE-cdh3 expressing cells in 20 nM paclitaxel (N = 49, mean = -25, *p* = 0.71). (**B**) Summary of collisions of ΔE-cdh3 expressing cells in 20 nM jasplakinolide (N = 37, mean = − 22.9°, *p* = 0.09). (**C**) Summary of collisions of WT cells in 20 nM nocodazole (N = 38, mean = 30.7°, *p* = 0.19). (**D**) Summary of collisions of WT cells in 20 nM cytochalasin D (N = 42, mean = 7.1°, *p* = 0.87). (**E**) The quantified directionality of single-cell migration of ΔE-cdh3 cell in 20 nM Paclitaxel (ΔE in Pac), ΔE-cdh3 cell in 20 nM Jasplakinolide (ΔE in Jas), WT cell in 20 nM Nocodazole (WT in Noc), and WT cell in 20 nM Cytochalasin D (WT in CytD) compared with ΔE-cdh3, and WT, respectively (light red for comparison from Fig. 2E). (**F**) Model of the hypothetical mechanism regulating CIL and directionality of single-cell migration in cells expressing non-junctional Cdh3 or truncated cdh3s through localized Rac1 activity and cytoskeleton stability. Rac1 is regulated independently by extracellular and cytoplasmic domains of non-junctional cadherin oppositely through unknown factors. Signals induced by cell–cell contact may also regulate CIL with potential signaling through either cis- and trans-bonds between Cdh3. In WT cells, Rac1 activity is held at moderate levels by the extracellular and cytoplasmic domains of non-junctional cdh3. Moderate Rac1 activity leads to moderate levels and gradient of cytoskeletal stability. As a stable cytoskeleton supports persistent migration, the moderate stability of the cytoskeleton in WT cells results in the moderate frequency of change in the direction of cell migration and enables CIL and moderate directionality of single-cell migration. In ΔE-cdh3 expressing cell, expression of the cytoplasmic domain of Cdh3 suppresses Rac1 activity and destabilizing the cytoskeleton; resulting in a low frequency of change in the direction of cell migration, defective-CIL, and lowered persistence. Meanwhile, ΔC-cdh3 hyper activates Rac1 and leads to the stable cytoskeleton and high frequency of change in the direction of cell migration, resulting in frequent turning and cells capable of CIL. The illustration was drawn using Adobe Illustrator Version 24.1.1 (https://www.adobe.com/products/illustrator.html).

Domain-dependent NJCad signaling through Rac1 might regulate CIL and cell directionality by modulating the dynamics of either the microtubule or F-actin cytoskeletal networks^45, 52^, implying that cytoskeletal destabilization is the cause of defects in CIL in ΔE-cdh3 expressing cells. To test this hypothesis, we modulated cytoskeletal stability in ΔE-cdh3 expressing cells, increasing the stability of microtubules with paclitaxel (a microtubule stabilizer) and F-actin with jasplakinolide (an actin filament stabilizer). Low doses of either cytoskeletal stabilizer partially rescued the defect in CIL and reduced the high directionality caused by ΔE-cdh3 (Fig. 5A,B,E; N = 49, mean = − 25°, *p* = 0.71, b; N = 37, mean = − 22.9°, *p* = 0.09 and for directionality E, and Table S2). Conversely, we observed that low doses of both nocodazole (microtubule destabilizer) and cytochalasin D (actin filament destabilizer) lowered CIL and increased directionality of WT cells (Fig. 5C–E; N = 38, mean = 30.7°, *p* = 0.19, d; N = 42, mean = 7.1°, *p* = 0.87 and for directionality E; see Table S2).

## Discussion

Classical cadherins function as cell surface receptors to provide sites for cell–cell adhesion, mechanically integrate the actin cytoskeleton across a tissue, and signal the presence and mechanical status of cell–cell contacts; however, our work on C-cadherin (Cdh3) in *Xenopus laevis* supports an emerging view that cadherins can signal in the absence of cell–cell contacts. This non-junctional role for Cdh3, revealed by expression of truncation mutants of Cdh3 indicates that non-junctional signaling mediates differential regulation of Rac1 activity through extracellular and cytoplasmic domains that modulate both CIL and the directionality of single-cell migration through cytoskeletal stability.

Based on our results, we propose a model in which the non-junctional extracellular and the cytoplasmic domain of Cdh3 control Rac1 activity in opposite ways, altering cytoskeletal stability to regulate CIL and the persistence of cell migration (Fig. 5F). Control over the stability of the cytoskeleton could allow NJCads to modulate the frequency of changes in the direction of cell migration; for instance, rapid turn-over of the cytoskeleton would tend to destabilize cell polarity, and thus limit persistent cell migration. Thus, the role of NJCads in regulating both CIL and cell migration directionality depends on the ability of WT cadherin to both activate and inhibit Rac1 activity. Since Rac1 is well known for its role in driving persistent cell migration and collective migration as well as its ability to modulate the stability of the cytoskeleton, this role for NJCads is consistent with current views on cell motility and collective migration^41, 45, 53, 54^.

Our model for the non-junction role of Cdh3 may also involve the formation of lateral cadherin-cadherin bonds on the same cell surface (cis-cadherin binding) since cis-binding can also alter biophysical properties of the cell^55^. For instance, mutant N-cadherin that cannot form cis-bonds showed a 10% decrease in Rac1 activity. By contrast, cis-bonds of E-cadherin are thought to be relatively weak and require trans-cadherin bonds (e.g. cell–cell adhesion)^56^. While we suspect the contribution of cis-binding is small in single isolated cells, its contribution to NJCad signaling through Cdh3 and its role in CIL remains to be tested. We also note that since endogenous levels of Cdh3 remain in our studies there may be continuing junctional-signaling between cells that make contact, both in vivo and during CIL.

Mutant cadherins, including intracellular and extracellular truncated forms developed over 20 years ago, have been used extensively to explore the role of cell–cell adhesion during *Xenopus* development. Here, we set out to use ΔE- and ΔC-cdh3^36^ to explore the role of Cdh3 during contact inhibition of locomotion in *Xenopus* mesendoderm. This choice was based on the broad adoption of extracellular and intracellular truncation mutants as pan- and type-specific dominant negative effectors, respectively, of classical cadherin function and cell adhesion (e.g.^11, 12, 36, 57–60^). Similar truncation mutants have been developed and broadly applied to investigate classical cadherin function during development and in disease processes involving cell–cell adhesion. Many additional cadherin mutations have been identified from their role in inheritable genetic disorders (e.g.^61^) and human disease^62^. In light of our results, we suggest a re-evaluation of cadherin mutants, including truncation, domain-swapped, and single-point mutants for their potential non-junctional roles; in cases where defects in collective cell migration or tissue-morphogenesis are observed we strongly encourage the use of model systems where studies of both collective and single-cell behaviors can be compared to help resolve non-junctional roles.

The precise molecular mechanism connecting non-junctional Cdh3 domains to CIL is still unknown. However, our results provide novel insights into the role of NJCads in regulating CIL and migration persistency. We propose NJCads regulate cell motility by regulating cytoskeletal stability through coordinately activating or suppressing Rac1 activity. Our study demonstrates that Cdh3 has non-junctional roles in collective migration and raises the question of how non-junctional signaling integrates with junctional signaling because cadherin engagement is known to regulate RhoA and Rac1 activity^7, 37–39^. We suspect some cadherins remain non-junctional even after cell–cell contact is established; for example, such coordination may underlie cell responses during cohesotaxis^63, 64^ where a pulling force on one side of the cell transmits a signal to initiate a protrusion on the opposite side. Such coordination could reflect the operation of simultaneous junctional and non-junctional cadherin signaling. Resolving these mechanisms will be aided by the development of live imaging tools able to dissect the dynamic movement and activity of cadherins involved in junctional as well as non-junctional signaling.

From our studies of mesendoderm collective migration, we propose a broader role for Cdh3 that integrates mechanosensing by junctional cadherins and signaling by NJCads to modulate Rac1 activity, cytoskeleton stability, and collective cell migration. Further studies are needed to map out molecular mechanisms of NJCads signaling and may provide insights into processes where multiple cadherin types are co-expressed or swapped, for instance, during mesenchymal-to-epithelial phenotypic transitions.

## Materials and methods

### *Xenopus laevis* embryos and constructs

*Xenopus laevis* embryos were obtained by in vitro fertilization using standard protocols^65^. Fertilized eggs were de-jellied in 2% cysteine solution (pH 8) and were microinjected at the one- or two-cell stage at multiple sites with a total of 0.5 ng (for FRET biosensors) or 1 ng (for other mRNAs) of mRNAs in 1 × modified Barth's Solution (MBS) containing 4% Ficoll-400 (17-0300-05, GE Healthcare) using a microinjector (PLI-90, Harvard Apparatus)^66^. Embryos were transferred to 1/3 × MBS and cultured until mid-gastrula stage (st. 11.5). All *Xenopus laevis* frogs used in this study were wild type pigmented and obtained commercially (Nasco, Fort Atkinson, WI).

Deletion mutants of the extracellular or cytoplasmic domain of Cadherin3, ΔE-Cdh3, and ΔC-Cdh3, constructed from the full length of Cdh3, were kind gifts from T. Kurth and P. Hausen^13, 36^. Based on consensus, we choose to use the name Cdh3 rather than former name C-cadherin for this *Xenopus laevis* gene. We note that Xenopus cdh3 is also known as P-cadherin in human and mammals. Cdh3 is the only classical cadherin present in the early gastrula; E- and N-cadherin, Cdh1 and Cdh2, respectively, are not found until later stages^67, 68^. The Cdh3 constructs used in this study are based on Cdh3.S, and not Cdh3.L, another variant, that has also been known by the name C-cadherin. Our Rac1 morpholino antisense oligonucleotide (RacMO; Gene Tools) was designed against the translational initiation site of *Xenopus laevis* Rac1 (5′-CCACACATTTAATGGCCTGCATGGC-3′). The effectiveness of RacMO-mediated knock-down was confirmed by western blot and showed that 12 ng total injection reduced Rac1 protein levels (Fig. S4A, C)^46^.

### Imaging conditions and quantitative methods of collective and single cell migration

To assess single and collective cell movements including CIL, we recorded live-cell movements using confocal microscopy, previously established for tracking *Xenopus* gastrula stage mesendodermal cells, and explants expressing membrane-targeted GFP^27^. For intravital, explant, and single-cell preparations, we first microsurgically removed a large patch of ectoderm from the animal pole region of a stage 11.5 embryo, exposing cells at the margin of the mesendoderm mantle. For intravital imaging, we placed the minimally exposed mesendoderm mantle and the remainder of the embryo onto a fibronectin-coated glass coverslip (Fig. 1B). This configuration allowed the mesendoderm mantle, including leading-edge cells, to contact fibronectin along with the same tissue interface they would encounter in vivo^27, 69^. In cases where the mesendoderm mantle closure is limited (e.g., ΔE-cdh3), we recreated tissue-scale collision by culturing two individual pieces of mesendoderm mantle on fibronectin-coated glass, so the leading edges of the two pieces would spread and make contact^27^. Confocal time-lapse sequences were acquired at 15-s intervals with a high N.A. 63 × oil immersion objective using an inverted confocal microscope (TCS SP5, Leica). Migrating cells were tracked, and their motion quantified using the Manual Tracker plug-in of ImageJ (https://rsb.info.nih.gov/ij/plugins/track/track.html) and analyzed with Excel (2010, Microsoft). To quantify mesendoderm closure, we calculated the distance between the rim at the start point and at each subsequent time point. Time-to-close graphs aligned individual trajectories from different experiments to the same closure time.

To assay single-cell migration and contact dynamics, we recorded the movement of mesendoderm cells dissociated from mesendoderm mantles prepared as described above. Dorsal mesendodermal cells were isolated at stage 11.5 in Ca^2+^–Mg^2+^-free DFA (Danilchik's for Amy; 53 mM NaCl, 5 mM Na_2_CO_3_, 4.5 mM K Gluconate, 32 mM Na Gluconate) and transferred into low Ca^2+^–Mg^2+^-DFA (0.2 mM Ca^2+^ and 0.2 mM Mg^2+^) in a small glass-bottomed chamber coated with fibronectin. Low Ca^2+^ and Mg^2+^ DFA allowed mesendoderm cells to migrate and collide but not aggregate as they would in standard media containing 1 mM Ca^2+^ and 1 mM Mg^2+^. Varying the concentration of Ca^2+^ and Mg^2+^ in single and collective cell experiments did not affect the directionality of single-cell migration (data not shown). Cell movements were recorded at 15-s intervals with a bright-field inverted microscope (Axiovert S100, Carl Zeiss) equipped with a digital camera (CFW-1308M, Scion Corporation). The following inhibitors were used: NSC23766 (733767-34-5, Santa Cruz Biotechnology), paclitaxel (T7191, Sigma-Aldrich), jasplakinolide (42017, Calbiochem), nocodazole (487928, Calbiochem), and cytochalasin D (C8273, Sigma-Aldrich).

CIL of single cells was quantified by established methods^24, 33^. Each cell collision was tracked with the Manual Tracking ImageJ plug-in. The location of cell–cell contacts and cell positions, 5 min prior to- and after-contact were analyzed. CIL parameters of angle and the ratio of the velocity before and after the collision were calculated and plotted using MATLAB (2012a, Mathworks). Statistical analysis performed with Excel calculated the mean angle and circular p-value (Rayleigh test; Table S1)^70^. The mean angle was measured with respect to the incident angle of the approaching cell. Normal CIL was defined in collisions, where the mean angle was significantly greater than 120° (*p* < 0.05), and defective-CIL was defined by collisions, where the mean angle was significantly less than 60° (*p* < 0.05). To quantify directionality, we tracked single cells that migrated without contacting other cells for one hour, using the Manual Tracking ImageJ plug-in. Cell directionality (start-to-end distance divided by path length) was calculated with Excel.

CIL of single cells colliding with beads used Cdh3-coated beads prepared by binding purified Cdh3-fc (gifted from Dr. Douglas W. DeSimone and Dr. Barry. M. Gumbiner) to Protein A/G coated beads (53132, Thermo Fisher Scientific)^71^. Briefly, twofold excess of Cdh3-fc needed to saturate the beads were incubated in 10 µM HEPES, 50 mM NaCl, pH 7.2, 1 mM CaCl_2_ for 90 min at 4 °C on an Eppendorf shaker (1,400 rpm). Cdh3-coated beads were pelleted, washed twice, and resuspended in 400 µl of 10 mM HEPES, and 50 mM NaCl, pH 7.2. Cadherin conjugation was confirmed by western blot using anti-cdh3 antibody (6B6, Developmental Studies Hybridoma Bank) and Odyssey CLx Imaging System (9140, LI-COR). Polyacrylamide beads (1504150, BIO-RAD) were used as controls.

### Rac1 activity and western blotting

Bulk Rac1 activity was measured using an assay kit (BK035, Cytoskeleton), as previously described^72–74^. Embryos were dissected and broken up in lysis buffer (50 mM Tris pH 7.5, 10 mM MgCl_2_, 0.5 M NaCl and 2% Igepal) with 1/100 (v/v) protease inhibitor cocktail (contained in the kit), 1/100 (v/v) phosphatase inhibitor cocktail 2 (P6726, Sigma Aldrich), 1/100 (v/v) phosphatase inhibitor cocktail 3 (P0044, Sigma Aldrich), and 25 mM sodium fluoride (919, Sigma Aldrich) to inhibit GTP hydrolysis^75^. The lysates were centrifuged at 14,000 *g* for 10 min × 5 times to remove lipid. The supernatant was used in a pull-down assay with PAK-PBD beads that bind active Rac1, and the products were run on a conventional protein blot. In brief, products were denatured by the addition of the same volume of 2 × SDS sample buffer (0.5 M Tris-HCl; pH 6.8, 10% (w/v) SDS, 50% (w/v) glycerin, 5% (v/v) 2-mercaptoethanol). Samples were then boiled for 5 min and processed by SDS-PAGE, blotted onto a PVDF membrane (162-0177, BIO-RAD), reacted with the 1:1000 anti-Rac1 monoclonal antibody (610651, BD Transduction Laboratories), and detected using 1:5000 IRDye 800CW anti-mouse antibody (926-32351, LI-COR) and Odyssey CLx Imaging System (9140, LI-COR). The expression level of the Cdh3 and Cdh3 mutant proteins was measured in the same protocol of western blot used for measuring Rac1 activity. Protein extracts were as described previously^76^. After transfer, blots were probed with mouse anti-cdh3 (C-cadherin; Clone 6B6, Developmental Studies Hybridoma Bank, dilution 1:300), mouse anti-cdh1 (E-cadherin; Clone 5D3, Developmental Studies Hybridoma Bank, dilution 1:300), anti-Myc (Clone 9E10, Sigma-Aldrich, dilution 1:1,000), and rabbit anti-ɣTubulin (T3320, Sigma-Aldrich, dilution 1:3,000). Secondary antibodies were IRDye 680LT Goat anti-Mouse IgG (Licor 925-68020) and IRDye 800CW Goat anti-Rabbit IgG (Licor 925-32211). Anti-myc was used to detect FL-cdh3, ΔC-cdh3, and ΔE-cdh3. Anti-cdh3 was used for FL-cdh3 and ΔC-cdh3. Anti-ɣtubulin was used for loading controls.

### Imaging subcellular dynamics of Rac1 using FRET probe and the analysis

To visualize Rac1 activity in living cells, we adapted a Raichu-Rac FRET probe (gift from Dr. Erez Raz)^48^. The original FRET sensor did not localize to the plasma membrane in Xenopus cells. To improve membrane association, we replaced the C-terminal CAAX domain with one from the H-Ras C-terminal domain (Fig. S5A). The modified Raichu-Rac probe showed clear membrane localization, significantly enhanced FRET signal at the membrane, and reduced non-specific activity in the cytoplasm (Fig. S5B). Time-lapse sequences of the FRET signal at 15-s intervals were acquired from an inverted confocal microscope (TCS SP5, Leica) using spectral detection of the YFP channel (535 ± 15 nm) and CFP channel (480 ± 20 nm) with excitation from the 458 nm argon laser line.

The FRET signal was calculated using previously published protocols^48, 49^. In brief, YFP and CFP channels of the Raichu-Rac1 FRET signal were individually processed by smoothing and background corrected. The FRET ratio image ([YFP]/[CFP]) was calculated using ImageJ. The signal intensities at the cell boundaries were calculated using custom MATLAB scripts at 360 points with a 10-point moving average (Fig. 4A–C, codes are available upon request).

The number of CIL events (in Fig. 4M) was counted when the direction of cell migration significantly changed within 5 min after the contact. Rac1 activity along a cell perimeter was categorized as “high” when the FRET ratio was greater than 1.2 and “low” when the ratio was less than 1.2.

## Supplementary information

Supplementary Information.

Supplementary Movie 1.

Supplementary Movie 2.

Supplementary Movie 3.

Supplementary Movie 4.

Supplementary Movie 5.

Supplementary Movie 6.

Supplementary Movie 7.

Supplementary Movie 8.

Supplementary Movie 9.
