# Supplementary material for: Non-junctional role of Cadherin3 in cell migration and contact inhibition of locomotion via domain-dependent, opposing regulation of Rac1
Source: Sci Rep. 2020 Oct 15;10:17326. doi: 10.1038/s41598-020-73862-y (PMC7567069; doi:10.1038/s41598-020-73862-y)
Supplement: Supplementary file 1 — Supplementary Information. [file 41598_2020_73862_MOESM1_ESM.pdf]

Supplementary information

Non-junctional role of Cadherin3 in cell migration and contact inhibition of locomotion via domain-dependent, opposing regulation of Rac1

Takehiko Ichikawa[1,2], Carsten Stuckenholz[1], and Lance A. Davidson[1,3,4*]

[1]Department of Bioengineering, University of Pittsburgh, Pittsburgh, PA, 15260, USA,
[2]Nano Life Science Institute (WPI-NanoLSI), Kanazawa University, Kanazawa 920-1192, Japan,
[3]Department of Developmental Biology, University of Pittsburgh, Pittsburgh, PA, 15260, USA,
[4]Department of Computational and Systems Biology, University of Pittsburgh, Pittsburgh, PA, 15260, USA

* Author for correspondence
Lance A. Davidson
Departments of Bioengineering and Developmental Biology,
University of Pittsburgh,
3501 Fifth Avenue, 5059-BST3,
Pittsburgh, PA, 15260, USA
Tel: +1-412-383-5820
Fax: +1-412-383-5819
E-mail: lad43@pitt.edu

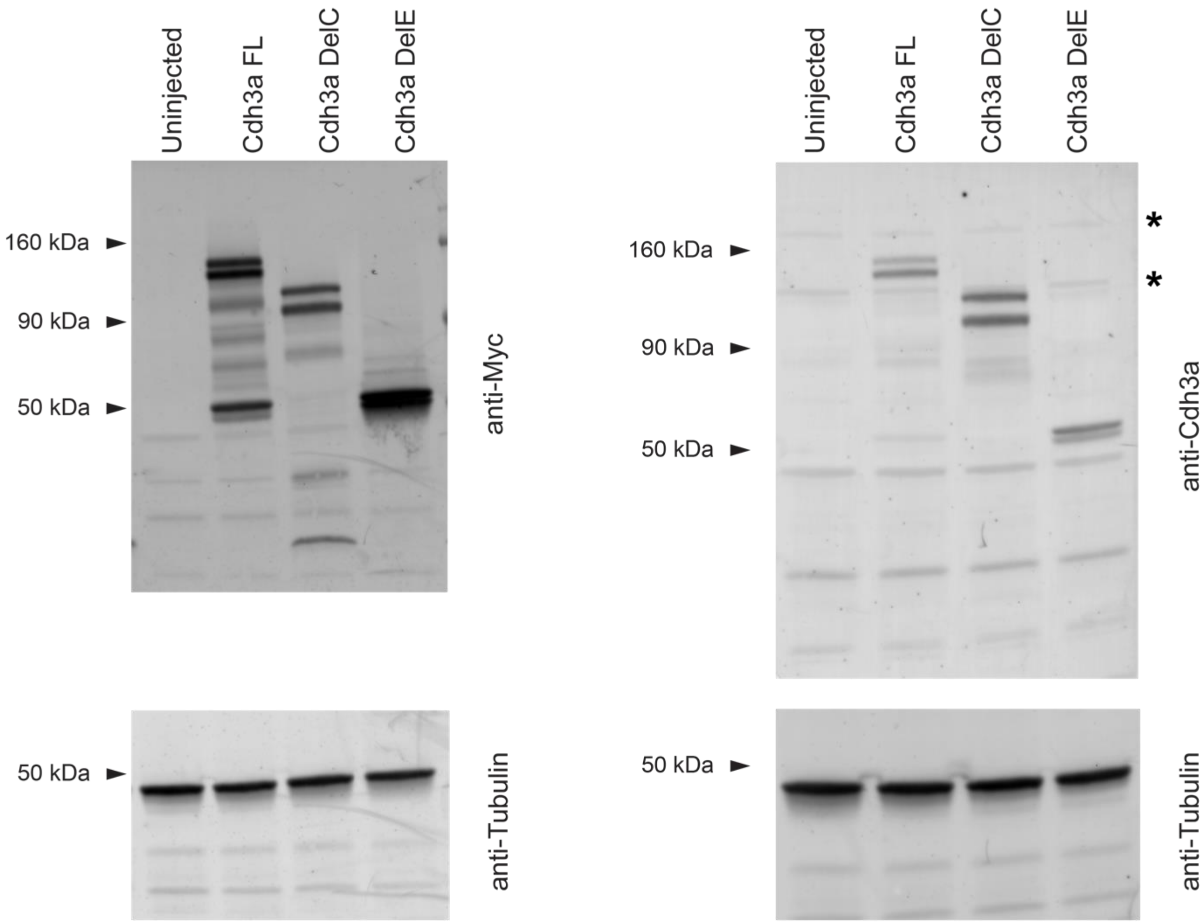

Figure S1. The expression level of FL-cdh3, ΔE-cdh3, and ΔC-cdh3. Western blot analysis of uninjected, FL-cdh3, ΔE-cdh3, and ΔC-cdh3 injected embryos using anti-Myc or anti-cdh3a compared with tubulin. Isoforms of endogenous Cdh3 are indicated by asterisks[86,87].

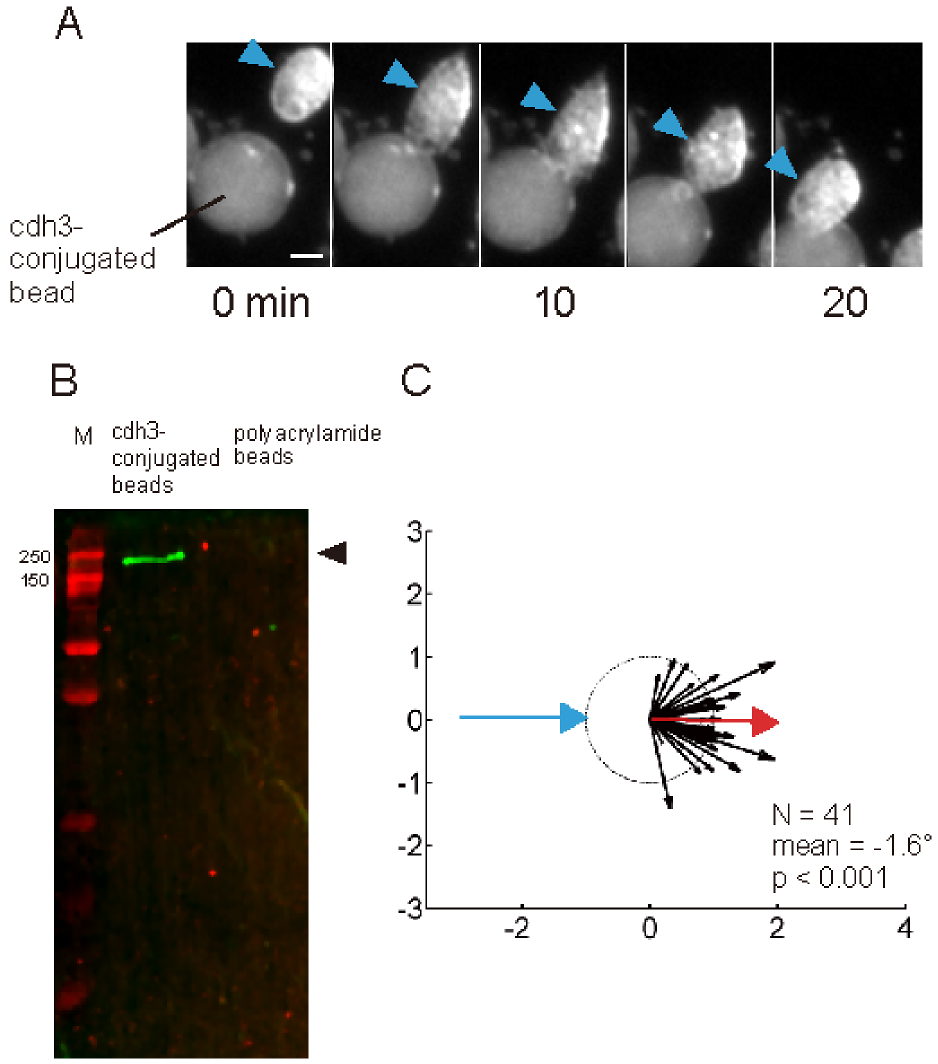

Figure S2. Collisions to cadherin3 conjugated beads. (A) Time sequence of the collision between a WT mesendodermal cell and a cdh3-conjugated bead. (B) Western blot analysis of cdh3-conjugated beads (Protein A/G beads) and control beads (polyacrylamide beads). M indicates the protein ladder marker. The position of cdh3 is indicated by the arrowhead. (C) Summary of collisions between WT cells and cadherin3-conjugated beads (N = 37, mean = 7.6°, $p < 0.001$). Scale bar: 20 μm.

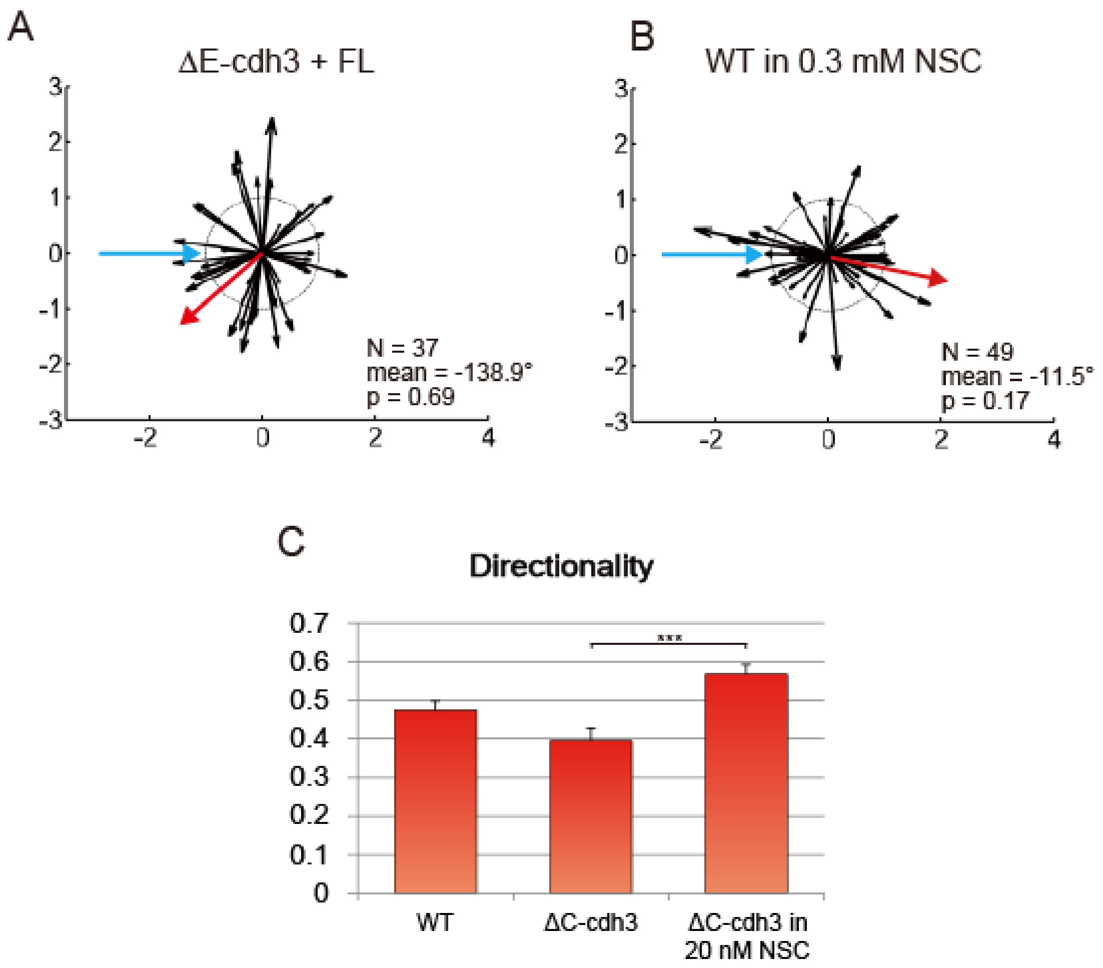

Figure S3. Single-cell CIL and single-cell persistence under various conditions. (A) Summary of collisions of ΔE-cdh3 + FL-cdh3 (N = 37, mean = -138.9, p = 0.69). (B) Summary of collisions of WT in 0.3 mM NSC23766 (N = 49, mean = -11.5, p = 0.17). (C) Low persistence of ΔC-cdh3 expressing cells is enhanced by Rac1 inhibition by 20 nM NSC23766.

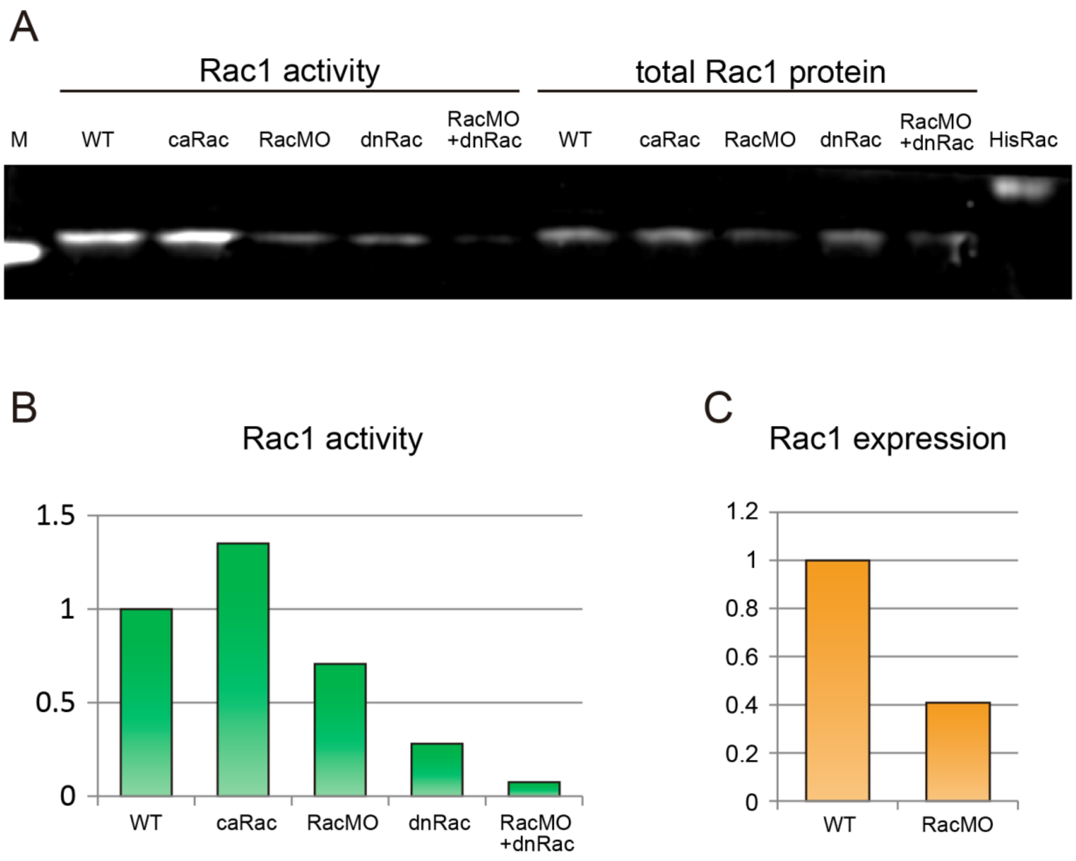

Figure S4. CaRac, RacMO, and dnRac alter Rac1 activity; RacMO reduces Rac1 abundance and activity. (A) Blot showing Rac1 activity and total Rac1 expression. M: marker band of 20 kDa, WT: wild type control, HisRac: His-tag conjugated purified Rac1 protein (24 kDa). (B) Quantification of Rac1 activation from (A) normalized to WT activity. The relative Rac1 activity after expression of caRac, RacMO, dnRac and RacMO + dnRac was 1.34, 0.7, 0.28, and 0.07 respectively. Full-length blots are presented in Figure S7B (C) The comparison of Rac1 protein expression between WT and RacMO from (A) normalized to WT expression levels. 12 ng injection of RacMO reduced Rac1 protein expression to 40% of WT levels.

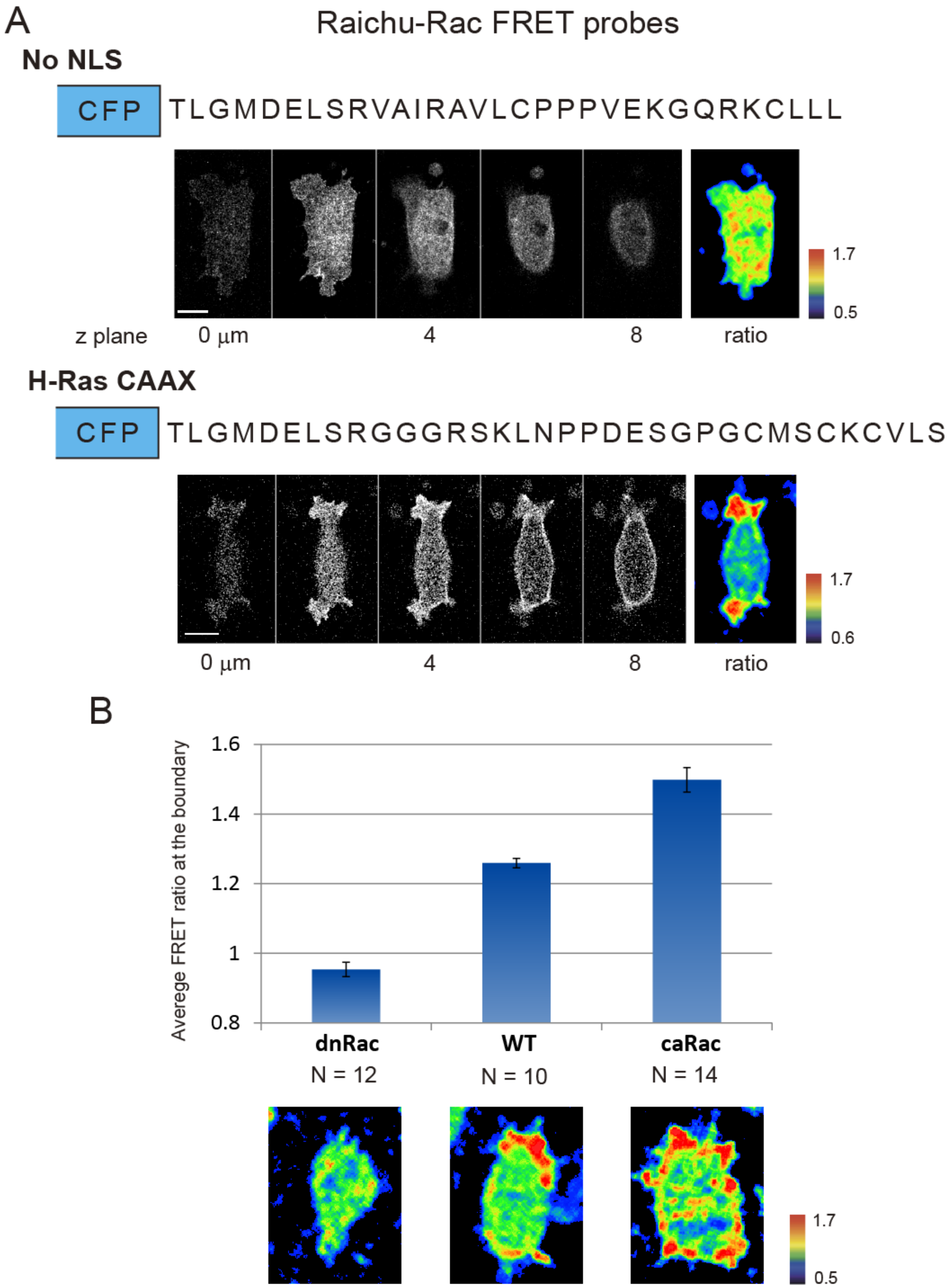
A
Raichu-Rac FRET probes
No NLS
CFP
TLGMDELSRVAIRAVLCPPPVEKGQRKCLLL
1.7
0.5
z plane
0 µm
4
8
ratio
H-Ras CAAX
CFP
TLGMDELSRGGGRSKLNPPDESGPGCMSCKCVLS
1.7
0.6
0 µm
4
8
ratio
B
Averege FRET ratio at the boundary
1.6
1.4
1.2
1
0.8
dnRac
N = 12
WT
N = 10
caRac
N = 14
1.7
0.5

Figure S5. Modified Raichu-Rac FRET biosensor with improved membrane localization. (A) Comparison between Raichu-Rac without the nuclear localization signal (no NLS)[46] and new Raichu-Rac with C-terminal domain of H-Ras (H-Ras CAAX). The top panel shows the amino acid sequences of C-terminal domain, slices from a z-series confocal stack of the YFP channel and the ratio of no NLS. The bottom panel shows FRET signal from H-Ras CAAX modified biosensor and clear polarity of Rac1 activity. (B) The response of Raichu-Rac with H-Ras CAAX in cells expressing dnRac and caRac. The FRET ratio value was calculated by averaging the FRET signal along the cell periphery over 10 minutes.

A

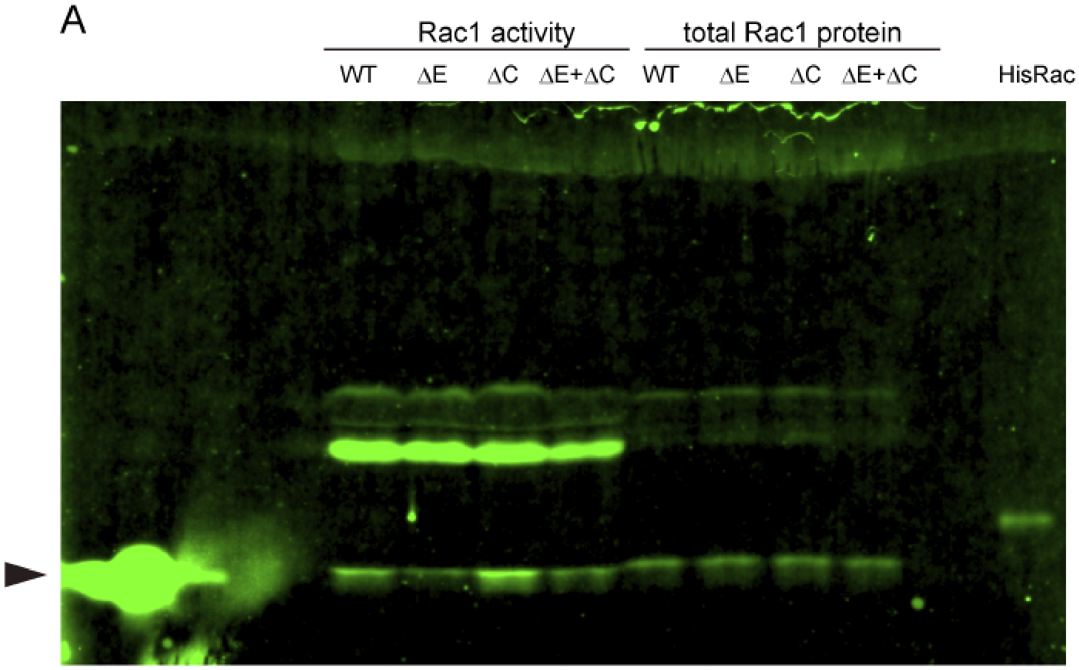

B

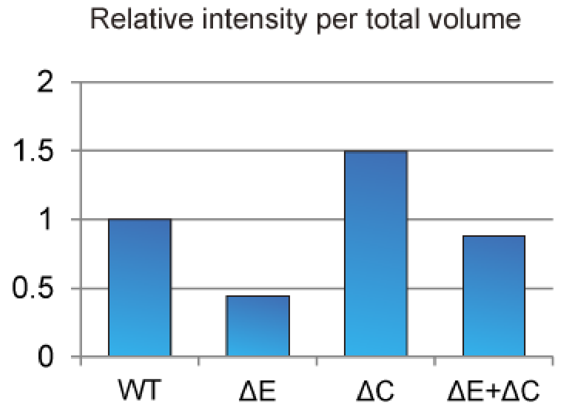

Figure S6. An additional blot of Rac1 activation of wild type and mutant cadherin injected embryo shown in Fig. 3A and B. (A) Original blot of additional Rac1 activation analysis in WT, ΔE-cdh3 (ΔE), ΔC-cdh3 (ΔC), and ΔE-cdh3 + ΔC-cdh3 (ΔE + ΔC) injected embryos. An arrowhead indicates the position of the Rac1 bands. (B) Rac1 activity of (A) normalized to WT levels.

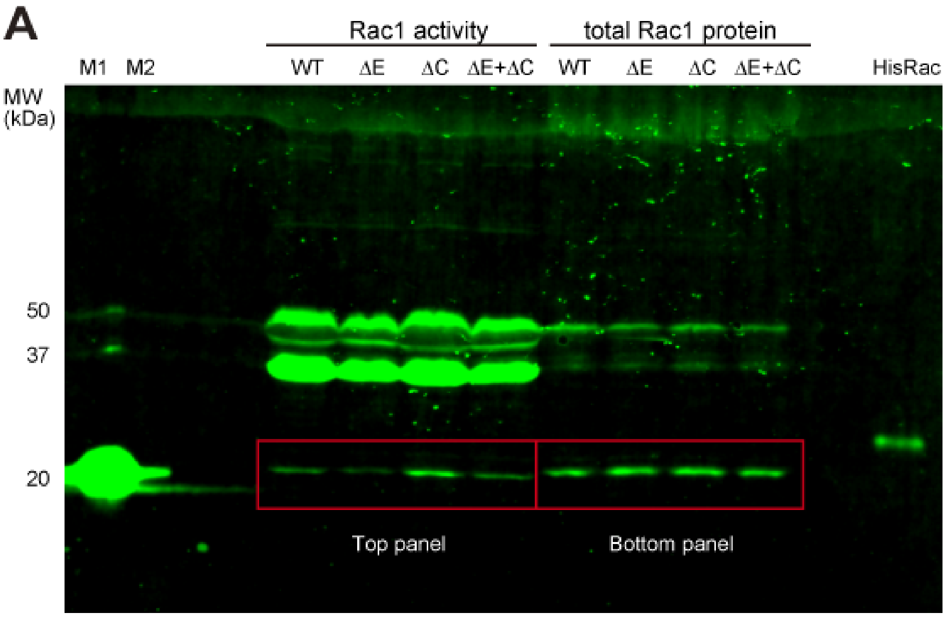

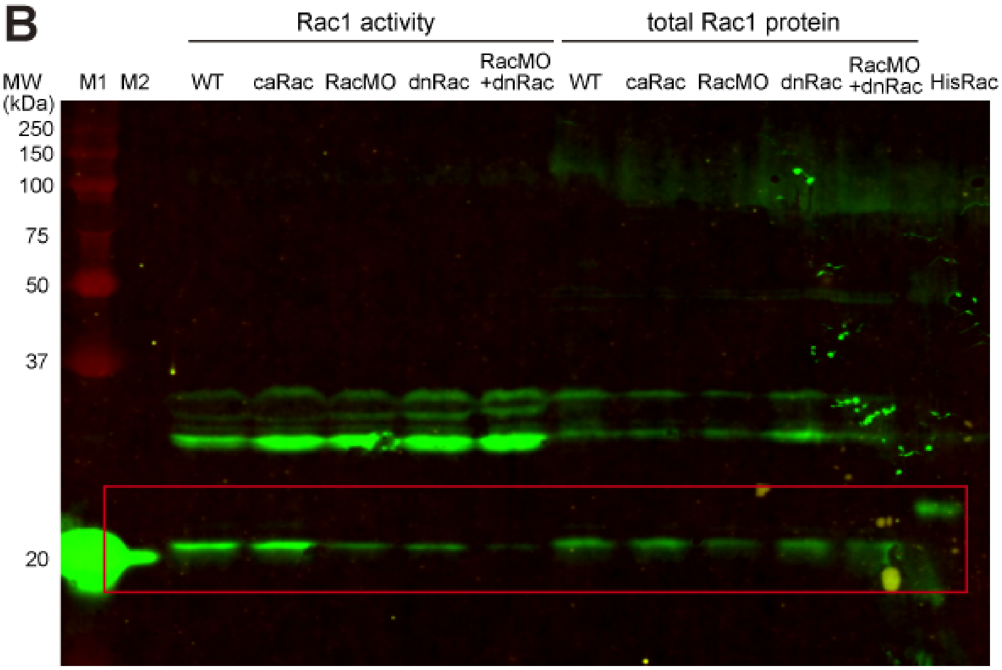

Figure S7. Original scans of western blot analyses presented. (A) Original scan of western blot in Figure 3A. M1: marker (Precision Plus Protein Standards, BIO-RAD), M2: 1/100 diluted M1 (volume/volume). Cropped regions are indicated with a red rectangle as appropriate. (B) Original scan of western blot in Supplementary Figure S4A.

Sample	N	mean (degree)	circular p	decision
WT	46	122.5	< 0.05	normal CIL
ΔE-cdh3	37	7.6	< 0.001	deficient
ΔC-cdh3	40	-179.8	< 0.01	normal
ΔE-cdh3 + ΔC-cdh3	47	-113.5	0.07	partial rescue
ΔE-cdh3 + FL-cdh3	37	-138.9	0.69	partial rescue
ΔE-cdh3 + caRac	42	-167.8	0.43	partial rescue
dnRac + RacMO	44	9	0.08	partial deficient
WT in 0.3 mM NSC23766	49	-11.5	0.17	partial deficient
ΔE-cdh3 in 20 nM paclitaxel	49	-25	0.71	partial rescue
ΔE-cdh3 in 20 nM jasplakinolide	37	-22.9	0.09	partial rescue
WT in 20 nM nocodazole	38	30.7	0.19	partial deficient
WT in 20 nM cytochalasin D	42	7.1	0.87	partial deficient
ΔE-cdh3 to WT	32	-6.7	< 0.001	deficient
WT to ΔE-cdh3	29	143.2	< 0.001	normal
WT to cdh3-beads	41	-1.6	< 0.001	deficient

Table S1. Mean angle and incidence of single-cell CIL.

The number of samples, mean angle, the p-value of Rayleigh test, and classification of contact inhibition of locomotion (CIL) are shown. Mean angle was measured with respect to the incident angle of the approaching cell. Normal CIL was defined in collisions, where the mean angle was significantly greater than 120° ($p < 0.05$). Cases of deficient CIL occur where the mean angle was significantly less than 60° ($p < 0.05$). Other cases are qualified as partial rescue or partial deficiencies in CIL.

Conditions	N	directionality	SE	p-value of paired t-test (for sample)
WT	54	0.47	0.0239	
ΔE-cdh3	43	0.6	0.0313	< 0.01 (WT)
ΔC-cdh3	35	0.39	0.0327	< 0.01 (WT)
ΔE-cdh3 + ΔC-cdh3	47	0.47	0.0173	< 0.001 (ΔE-cdh3)<br>< 0.001 (ΔC-cdh3)
ΔE-cdh3 + FL-cdh3	37	0.46	0.0295	< 0.01 (ΔE-cdh3)
ΔC-cdh3 + FL-cdh3	36	0.43	0.032	< 0.05 (ΔC-cdh3)
ΔE-cdh3 + caRac	37	0.46	0.027	< 0.01 (ΔE-cdh3)
ΔC-cdh3 + dnRac	37	0.51	0.0282	< 0.001 (ΔC-cdh3)
caRac	41	0.38	0.0314	< 0.05 (WT)
dnRac + RacMO	37	0.61	0.0276	< 0.001 (WT)
ΔC-cdh3 in 20 nM NSC23766	49	0.56	0.0248	< 0.001 (ΔC-cdh3)
ΔE-cdh3 in 20 nM Pacritaxel	52	0.39	0.0186	< 0.001 (ΔE-cdh3)
ΔE-cdh3 in 20 nM Jasplakinolide	44	0.41	0.024	< 0.001 (ΔE-cdh3)
WT in 20 nM Nocodazole	51	0.53	0.0221	< 0.05 (WT)
WT in 20 nM Cytochalasin D	37	0.55	0.0283	< 0.01 (WT)

Table S2. Persistence (directionality) of single migrating cells.

The number of samples, directionality, standard error, and p-value of paired t-test for each sample in parentheses are shown. Values were collected by tracking single migratory cells for 1 hour without collision.

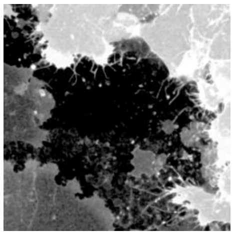

Movie 1. Mesendoderm closure within wild type embryo.
Intravital confocal time-lapse sequence of mesendoderm closure expressing membrane-targeted GFP. The different expression level of GFP indicates the cells originating from different regions around the marginal zone. Images were acquired every 15 seconds with a high N.A. oil-immersion 63× lens and an inverted confocal microscope. Scale bar is 20 μm.

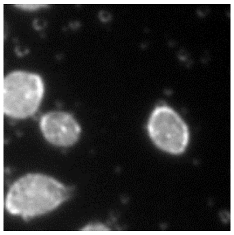

Movie 2. Single-cell CIL between wild type mesendoderm cells.
The time-lapse sequence shows CIL between single wild type mesendodermal cells observed using brightfield illumination on an inverted compound microscope. The time interval is 30 seconds. Scale bar is 20 μm.

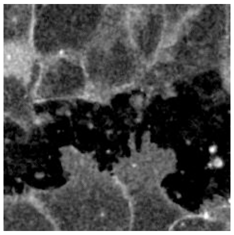

Movie 3. Mesendoderm closure of ΔE-cdh3 expressing embryo.
Confocal time-lapse sequence of mesendoderm closure expressing ΔE-cdh3 and membrane-targeted GFP. Data were acquired every 15 seconds. Scale bar is 20 μm.

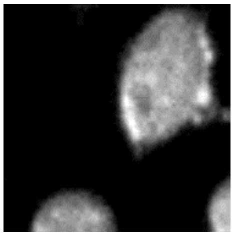

Movie 4. Single-cell CIL of ΔE-cdh3 expressing mesendoderm.
Time-lapse sequence of single cells expressing ΔE-cdh3 observed using brightfield illumination on an inverted compound microscope. The time interval is 30 seconds. Scale bar is 20 μm.

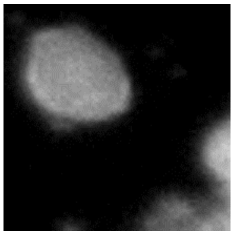

Movie 5. Single-cell CIL of ΔC-cdh3 expressing mesendoderm.
Time-lapse sequence of single cells expressing ΔC-cdh3 observed using brightfield illumination on an inverted compound microscope. The time interval is 30 seconds. Scale bar is 20 μm.

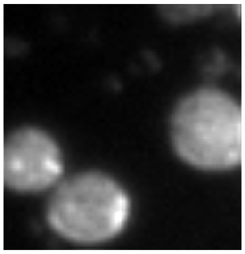

Movie 6. Single-cell CIL of ΔE-cdh3 + ΔC-cdh3 co-expressing mesendoderm.

Time-lapse sequence of single cells co-expressing ΔE-cdh3 and ΔC-cdh3 observed using brightfield illumination on an inverted compound microscope. The time interval is 30 seconds. Scale bar is 20 μm.

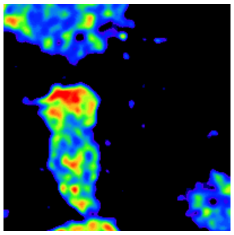

Movie 7. Subcellular Rac1 activity of wild type mesendodermal cell during a cell-cell collision.

Confocal time-lapse sequence of wild type single-cell CIL showing Rac1 activity before, during, and after the cell-cell collision. Data were acquired every 15 seconds with a 63× lens and an inverted confocal microscope. Scale bar is 20 μm.

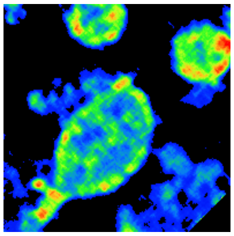

Movie 8. Subcellular Rac1 activity of ΔE-cdh3 mesendodermal cell during a cell-cell collision.

Confocal time-lapse sequence of ΔE-cdh3 single cell CIL showing Rac1 activity before, during, and after the cell-cell collision. Data were acquired at every 15 seconds. Scale bar is 20 μm.

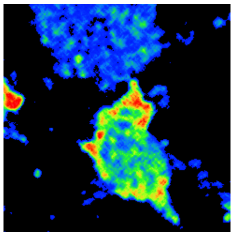

Movie 9. Subcellular Rac1 activity of ΔC-cdh3 mesendodermal cell during cell-cell collision.

Time-lapse movie of ΔC-cdh3 single-cell CIL showing Rac1 activity before, during, and after the cell-cell collision. Data were acquired every 15 seconds. Scale bar is 20 μm.
